# Digital therapeutics in neurology

**DOI:** 10.1007/s00415-021-10608-4

**Published:** 2021-05-20

**Authors:** G. Abbadessa, F. Brigo, M. Clerico, S. De Mercanti, F. Trojsi, G. Tedeschi, S. Bonavita, L. Lavorgna

**Affiliations:** 1 Division of Neurology, University of Campania Luigi Vanvitelli, Naples, Italy; 2 Department of Neurology, Hospital of Merano (SABES-ASDAA), 39012 Naples, Italy; 3 Clinical and Biological Sciences Department, University of Torino, 10124 Turin, Italy

**Keywords:** Digital therapeutics, Exergames, Neurological disorders, Rehabilitation, Validation studies

## Abstract

Digital therapeutics (DTx) is a section of digital health defined by the DTx Alliance as “delivering evidence-based therapeutic interventions to patients that are driven by software to prevent, manage, or treat a medical disorder or disease. They are used independently or in concert with medications, devices, or other therapies to optimize patient care and health outcomes”. Chronic disabling diseases could greatly benefit from DTx. In this narrative review, we provide an overview of DTx in the care of patients with neurological dysfunctions.

## Introduction

The term digital health is rooted in eHealth, defined as “the use of information and communications technology in support of health and health-related fields.” [1, 2]. It describes all technologies, platforms, and systems that engage consumers for lifestyle, wellness, and health-related purposes; capture, store or transmit health data; and/or support life science and clinical operations [1, 2]. Digital therapeutics (DTx) is a section of digital health defined by the Digital Therapeutics Alliance as “delivering evidence-based therapeutic interventions to patients that are driven by software to prevent, manage, or treat a medical disorder or disease. They are used independently or in concert with medications, devices, or other therapies to optimize patient care and health outcomes.” [3]. DTx could overcome several limits related to traditional clinical practice, reduce costs associated with attending hospital or doctor’s clinic, [4] improve adherence to healthy lifestyle behavior and prescribed medications [4] allow continuous monitoring, [4] and optimize the time of administrative tasks and routine communication [4]. Therefore, chronic disabling diseases could greatly benefit from DTx.

DTx tools include several screen devices such as smartphones, tablets, computers, and videogame platforms that converge with software algorithms [4] and that can be applied for improvement of therapy management and rehabilitation [4]. This narrative review provides an overview of DTx in the care of patients with neurological dysfunctions.

## Methods

We performed a comprehensive search of the medical literature using PubMed to identify DTx tools used in clinical practice for the treatment of neurological dysfunctions. To this aim, we used the following terms and keywords in different combinations: “digital therapeutics”, “videogame”, “telerehabilitation”, “exergame”, “serious game”, “virtual reality”, “neurorehabilitation”, “neurological disease”, neurological disorder”. Relevant articles were identified and located individually to examine citing and cited-by articles. We selected what we considered the most relevant DTx tools, in terms of novelty, ease of use, evidence from studies, clinical relevance particularly with regards to patient-important outcomes. Studies answering the aim of this review were selected and reported. For each DTx included we searched for the different language(s) that were available. We only included DTx devices that have proven their effectiveness and ease of use in clinical studies; for each DTx included we referred to published studies, particularly focusing on randomized controlled trials (whenever available). We provided a narrative description of these tools, addressing their role in the management and therapy of motor and sensory system symptoms, cognitive impairment, and patients’ compliance.

## Sensorimotor functions

Visual and proprioceptive inputs are required for accurate and adaptable motor control and the acquisition of motor skills, therefore intact sensory functions are critical components to facilitate motor rehabilitation [5–7].

The use of iPads, smartphones, and virtual reality software enables productive training with enhanced sensory stimulation [8].

Recent studies showed the potential of virtual reality-based interventions to benefit patients with upper extremities motor impairment, balance, and gait dysfunctions [8]. In recent years, several randomized controlled trials showed that virtual reality-interventions for motor rehabilitation are highly suitable for both upper and lower limb motor training [8]. Virtual reality-techniques can provide various virtual environments allowing interaction between virtual objects and motion [8]. The “Computer-Assisted Rehabilitation Environment” (CAREN) is an interactive-motion-based technology for post-stroke upper limb rehabilitation. In a randomized controlled trial [9] CAREN was compared with traditional physical exercise training. The training, simulating a supermarket shopping scene with visual tridimensional cues, engages the patient in a reach to grasp task. Patients performed the task in the sitting position to favor the full range of movement at the shoulder and elbow level. The training schedule for both groups was 45 min, 3 days/week for 4 weeks. Both trainings showed a significant improvement in arm motor impairment measures, clinical impairment scores, and activity levels post-intervention with a greater improvement for shoulder adduction and flexion and elbow extension in the intervention group. However, for some outcomes, the significant between-group difference was small. Moreover, although significant changes at the Reaching Performance Scale for Stroke and elbow subscale were detected, the absence of a significant clinical difference between the two groups does not allow to conclude for the superiority of CAREN compared to the traditional intervention. In this perspective, the digital intervention may be useful in those conditions that do not allow a traditional approach (i.e. during a pandemic, for unserved people, etc.) or in continuation to traditional therapy to consolidate the benefits.

“Leap Motion Controller”, an optical hand-tracking module that captures hand movements with submillimeter accuracy without the need to use wearable sensors or devices, generates a virtual image of the upper limbs on a screen and the patient is driven to perform movements according to the suggested functional task [10]. A feasibility study evaluated the effectiveness of the “Leap Motion Controller” system for upper limb rehabilitation in patients with Parkinson's disease [10]. The study results suggest an improvement in upper limb coordination, speed of movements, and fine dexterity using the “Leap Motion Controller” system [10].

There are few studies related to rehabilitation systems focusing on the improvement of proprioception. To improve proprioception, sensorimotor training programs have been suggested to facilitate joint position sense and dynamic joint stability using rhythmic active motion, angle repositioning, and standing on an air cushion with support to stimulate muscular co-activation [11]. Kim et al. investigated a new type of rehabilitation system to train proprioception of stroke patients using virtual reality technology [12]. The system requires that patients move their arm to the target position relying on proprioception feedback only. Repeating this procedure, stroke patients could adjust the proprioception of their arm. This study showed that virtual reality proprioception feedback training improved motor control in stroke patients [12].

Virtual reality is also a promising new alternative to traditional rehabilitation therapy for the management of balance problems [9]. Kalron et al. [13] evaluated the efficacy of a training program using the “CAREN” for improving balance in patients with multiple sclerosis. In a randomized controlled trial, 32 patients with multiple sclerosis were randomized into the virtual reality-based intervention group and the control group. Each group received balance training sessions for 6 weeks, twice per week, 30 min sessions. Both groups showed an improvement in clinical balance tests and posturography measures [13]. Moreover, the group using virtual reality-based DTx showed a greater improvement compared to the control group in the functional reach test and the fear of falling questionnaire [13].

Videogame-based rehabilitation programs for motor rehabilitation in patients with neurological diseases, called exergames, were also assessed in clinical studies [14–17]. Exergames are videogames that require physical exertion to play the game, regardless of their primary purpose, and could be done using virtual reality-based DTx devices [14]. They could address both upper and lower limb motor dysfunction as well as balance impairment. “Jintronix Rehabilitation System” (JRS WAVE), available in the English language, is a Microsoft-Kinect-based virtual reality software program delivering motor rehabilitation [16]. The system targets upper limb function, standing and sitting balance, and gait, using fun and engaging video games that can be played in both home and outpatient settings. In the randomized controlled trial [16] comparing virtual reality-based training under therapist supervision to standard physiotherapy, subjects in the intervention group used the device for 1 h/week for 8 weeks. Both the intervention and control groups showed an improvement in the standing balance (primary outcome), with no significant difference in any primary or secondary measures [16]. These results confirm that exergames could be a useful complement or alternative to traditional rehabilitation tools.

Yazgan et al. [17] investigated, in patients with multiple sclerosis, the effects of exercise training with two different exergaming systems (“Nintendo Wii Fit” and “Balance Trainer”) on balance, functionality, fatigue, and quality of life, in comparison with no intervention. The subjects in the intervention group received an exercise program under the supervision of a physiotherapist 2 days/week for 8 weeks. They revealed that the training with “Nintendo Wii Fit” and “Balance Trainer”, improved balance, increased functionality, reduced fatigue severity, and improved quality of life [17].

“Nintendo Wii Fit Board Balance” was also explored in two randomized clinical studies to evaluate its effectiveness in addressing balance impairment of patients with Parkinson's disease [18, 19]. Liao et al. [18] evaluated the effect of the tool on dynamic balance. Obstacle crossing is a balance-challenging task and can cause falls in people with Parkinson's disease. To examine the effects of virtual reality-based exercise on obstacle crossing performance and dynamic balance, patients with Parkinson's disease were randomized into three groups. In the exercise groups, participants received virtual reality-based Wii Fit exercise or traditional exercise for 45 min, followed by 15 min of treadmill training in each session for a total of 12 sessions over 6 weeks. Participants in the control group received no structured exercise program. Patients with Wii training showed a greater improvement in primary outcomes (obstacle crossing velocity, and dynamic balance) compared to the control group. Patients in Wii group also showed a greater improvement in movement velocity of the limits-of-stability test than patients undergoing traditional exercise [18]. Further evidence in support of this tool to address sensorimotor disability of patients with Parkinson's disease comes from a more recent clinical study that analyzed the effectiveness of exergaming with “Wii Balance Board” in improving functional balance, fatigue, functional exercise capacity, and quality of life [19]. Twenty patients were randomized into two groups: an exergaming group and a conventional exercise group. Patients in the intervention group underwent a 30 min exergaming session with prior practicing of the required postures and movements, 2 sessions per week for 12 weeks. Patients in the control group underwent traditional exercise for 30 min session, 2 sessions per week for 12 weeks. Exergaming was effective in enhancing balance and reducing fatigue in patients with Parkinson disease after 12 weeks of treatment. However, this benefit was not sustained in the long-term [19].

Beyond the relationship between sensory and motor function, many studies suggest a positive effect of physical activity and structured exercise on cognitive functions, with the most consistent findings being reported for executive functions [20].

Ozdogar et al. [21], in a recent randomized controlled trial, aimed to investigate in patients with multiple sclerosis the effect of video-based exergaming training on upper extremities and cognitive function and on other multiple sclerosis-related symptoms. Sixty patients were randomly divided into three groups: video-based exergaming, conventional rehabilitation, and control groups. The intervention groups received therapy sessions once a week for 8 weeks. The results of the study suggested that video-based exergaming is almost as effective as conventional rehabilitation regarding the improvement in walking, upper and lower limbs functions, cognitive functions, fatigue, depression, and health-related quality of life [21].

The “Interactive Rehabilitation Exercise Software” (IREX) is designed to address coordination and balance impairment and was applied in a blinded, parallel-group randomized controlled trial conducted in an inpatient stroke rehabilitation unit [22]. Participants in the treatment group interacted with the virtual reality games (eg, soccer goaltending, snowboarding) in a standing position, thereby challenging their balance and weight shifting. In contrast, individuals in the control group were seated and played games that did not require any weight shifting within their base of support. Participants in both groups completed 10–12 sessions of 20 min of interactive virtual reality exercise using IREX in addition to their regular inpatient rehabilitation therapy sessions. The primary outcome measure was the Timed Up and Go test. Secondary outcome measures included the Two-Minute Walking Test and the Chedoke McMaster Stroke Assessment Scale Leg domain.

Both groups met minimal clinically important difference values after the final training session for the Timed Up and Go test and the Two-Minute Walking Test. More individuals in the treatment than the control group showed improvements on the Chedoke McMaster Stroke Assessment Scale Leg domain after the final training session [22]. Results of this study suggest that virtual reality balance and mobility exercises might positively impact inpatient stroke rehabilitation, [22] but should be read with caution, due to an overlap in the confidence limits suggesting imprecision.

Concerning tablet application for motor rehabilitation, some tools are available. “FINDEX”, an Android-based tablet game, assesses and monitors a patient’s fine finger dexterity such as finger control, and coordination, and range of motions [23]. Stroke patients were trained in an in-patient setting using the application, and the result of the study showed improvement in dexterity in all patients. The findings from the study suggested that mobile games such as “FINDEX” may be used as an effective therapeutic tool for fine motor rehabilitation in a clinical setting [23]. Other iPad-based therapeutic tools developed to improve fine motor skills include “Fruit Ninja” and “Dexteria” [8]. Among the applications for tablets to train upper limb fine motor function a further approach for stroke patients is music therapy: patients are engaged with digital instruments to train fine motor skills; the underlying integration of the auditory-sensory motor circuit could improve speed and precision of movement [24]. An example is “MUSIC-SUPPORTED THERAPY”, which provides a series of exercises using electronic musical instruments and showed to improve upper limb function in a clinical study [24]. It is important to take into account that training must be followed by exercise, repetition, and practice; in this perspective, a main objective of the digital devices should be to improve patients’ compliance convincing them to continue motor training [8].

The Internet-based home training program “eTraining” was designed to target balance impairment [25]. A randomized controlled trial explored the effectiveness of “eTraining” compared to hippotherapy. Patients with multiple sclerosis received hippotherapy or Internet-based home training twice a week for 12 weeks. The study showed a comparable improvement in static and dynamic balance with both intervention programs [25]. Home-based technologies have also been used to increase physical activity through an Internet-delivered behavioral intervention and to provide Web-based physiotherapy exercises [26].

## Cognitive functions

Cognitive rehabilitation via digital devices is increasingly regarded as a potentially effective rehabilitative option to enhance brain neuroplasticity [27]. An example is “Cogmed”, an online platform providing therapeutic training to improve working memory. Cogmed was explored in two randomized controlled studies that showed a significant improvement in working memory and executive functions in the intervention group compared to the control group [28, 29]. “Constant Therapy” is an i-Pad based application to rehabilitate patients with speech, language, and cognitive deficits, caused by stroke, brain trauma, and other neurological diseases [30]. It provides tasks to train speech and memory. In a clinical controlled study, [30] “Constant Therapy” intervention was compared with a traditional approach. Both groups received 1-h clinic session with a clinician; furthermore, patients in the intervention group also received “Constant Therapy” at home for 1 h, once a week, for 10 weeks. “Constant Therapy” demonstrated to improve memory tasks in patients using the tool compared with patients who did not, showing the feasibility of using tablet-based software to deliver a tailored speech and cognitive therapy [30]. To date, virtual reality-based cognitive training is less explored in clinical studies compared to computer-based platforms and i-Pad applications. A recent randomized controlled trial showed a greater improvement in attention and memory skills in the group using virtual reality-based devices compared to subjects that underwent standard cognitive rehabilitation [31]. However, the small sample size (6 patients per group) and the short follow-up do not allow to generalize the findings to all stroke patients [31].

Aphasia rehabilitation encompasses semantic training, for patients with fluent aphasia, and phonological training, for patients with non-fluent aphasia [8]. Semantic and phonological training tools are available as computer-based interventions, tablet based-interventions, and virtual reality-based interventions [8]. Usually, both types of training are used in combination for aphasia rehabilitation [8]. “StepByStep” and “Multicue”, are computer-based word-finding therapies for stroke patients. They were assessed in two randomized controlled trials showing a significant improvement in naming abilities in patients who underwent the training [32, 33].

Semantic and phonological training tools for aphasia rehabilitation are available also for iPad. “Constant Therapy”, “Lingraphica Talkpath Therapy”, “Language TherAppy”, and “iBooks” are some examples of the iPad-based available tools evaluated in clinical studies [30, 34, 35]. “Constant Therapy” includes 37 evidence-based cognitive and language tasks and showed to improve speech accuracy and latency in stroke patients with aphasia [30]. “Lingraphica TalkPath Therapy” includes exercises that target writing, speaking, reading, and listening [34]. In a study, patients undergoing aphasia rehabilitation with this tool showed a significant improvement in spontaneous speech, repetition, naming tasks, and auditory-verbal comprehension [34]. “Language TherAppy” is a tablet-based self-administered speech therapy [35]. In a clinical trial, it showed to improve several language tasks, such as comprehension, naming, reading, and writing [35]. “iBook” is a tablet-based technology targeting communication deficits explored in a clinical study. All the participants undergoing a home intervention with “iBook” showed to maintain and augment the improvement obtained during the previous 2 weeks of intensive traditional speech and language therapy [36].

Additionally, phonological training programs are available in virtual reality-based modalities. “Sentactics®” is a computer-automated treatment for improving non-fluent aphasia. It provides a virtual clinician that guides patients during the training. In a clinical controlled study evaluating its efficacy, “Sentactics®” was found to be a feasible alternative to traditional therapy for aphasia rehabilitation programs [37].

Acquired writing impairment, or dysgraphia, is common in aphasia [38]. It affects both handwriting and typing and may recover less well than other aphasic symptoms [38]. A recent clinical trial evaluated the benefits of technology-enhanced writing therapy for people with acquired dysgraphia [38]. Twenty-one people with dysgraphia received an initial 1–2 h of technology training. This was followed by 12 1-h sessions of therapy delivered over 6 weeks. The primary outcome was the improvement of functional writing measures following therapy using assistive technology. Study results suggest that dysgraphia improved following therapy using assistive technology [38].

“Cognitive Training Kit” (COGNI-TRAcK) is a customized application software for self-administered intensive and personalized cognitive training at home. It is based on working memory exercises. In a first study, 16 patients with multiple sclerosis and cognitive impairment were submitted to an 8-week at-home intervention administered through the app. The intervention consisted of five 30-min sessions per week for 8 weeks. The application was found to be usable, motivating, and well-accepted [39]. A study on twenty-eight patients with multiple sclerosis evaluated the effectiveness of the “Cognitive Training Kit” comparing adaptive versus nonadaptive cognitive training [40]. The authors concluded that “Cognitive Training Kit” is a suitable tool for cognitive rehabilitation in multiple sclerosis patients and only with an adaptive working load is effective as cognitive training [40]. A recent pilot study was carried out to assess whether an in-home, video game-like tablet-based digital treatment is superior to a control word game in improving processing speed in adults with multiple sclerosis. Both interventions resulted in significant improvements in processing speed. Seventy percent of participants undergoing to the in-home digital intervention maintained a clinically meaningful improvement in processing speed at the end of the post-treatment observation period, compared with 37% of the participants randomized to the active placebo control group. The authors concluded that the in-home digital intervention resulted in substantial and durable improvements in processing speed [41].

There is also increasing evidence showing that motor exergames may prove beneficial for cognitive performances [42]. Exergames can positively impact several cognitive functions, such as memory, attention, and visuospatial abilities, as well as motor functions, such as balance and gait [43].

Few serious games (games designed for a primary purpose other than pure entertainment) specifically targeting patients with Alzheimer disease have been recently developed [44] “MINWii” (on Nintendo R WiiTM) is a serious game to administer active music therapy in which the player plays on a virtual keyboard a well-known song [45]. This is a renarcissization-based approach through the use of video-games and music therapy. Renarcissization aims to restore self-esteem in patients who have gradually grown to consider themselves as a useless burden for their caregivers. This approach provides patients with the opportunity to engage and enhance residual capabilities [45]. The goal is to improve patients’ self-image (renarcissization), to reduce behavioral symptoms, which are an important cause of institutionalization [45].

Kitchen and Cooking is a cooking tablet serious game: following a recipe, the player selects the right ingredients from the kitchen, plans the actions necessary to complete the recipe, and finally performs specific gestures to accomplish each action. The app targets executive functions, explicitly planning abilities but also includes activities to train attention and object recognition, as well as praxis [46]. A study on 21 patients (9 with mild cognitive impairment and 12 with Alzheimer disease) based on self-report questionnaires assessing their overall game experience, showed overall acceptability of this serious game, suggesting its efficacy to assess and stimulate executive functions (such as planning abilities) and praxis, also in apathetic patients [46].

“MeMo” is a Web-based app for memory, attention, and mental flexibility training. In a randomized controlled trial, its effects on cognitive and behavioral symptoms were explored in 46 patients with neurocognitive disorders (32 participants were diagnosed with probable Alzheimer's disease and 14 with mixed disorders) [47]. In detail, the study compared patients using and not using “MeMo” for 12 weeks (four sessions per week). The results showed a small but significant improvement in attention and apathy over a 3-month training period. However, these positive effects on attention and motivation were observed only with regular use of the app [47].

## Interaction between cognitive & motor functions

The mechanisms to explain the effects of physical exercise on cognition are far to be completely known, however, there may be some possible interpretation either at a cellular or at a behavioral level. At a cellular level, it has been shown that the mammalian brain exhibits persistent plasticity throughout all stages of life [48]. Neuronal plasticity allows to learn new skills, to consolidate and retrieve memories, to reorganize neuronal networks, particularly in response to environmental stimuli [49]. At a behavioral level, it is possible that, with practice, tasks become automatic and less demanding in terms of attention. Alternatively, continuous physical training may facilitate the development of less attention-demanding strategies. The amelioration of task performances after aerobic exercise concerns tasks involving executive control of attention. Moreover, it has been hypothesized that cardiovascular fitness may improve the efficiency of neural processes or may provide increased metabolic resources for task performance [50, 51].

Therefore, a combination of cognitive treatment and physical training could be more promising than interventions focused on only one domain for inducing stable improvement of different cognitive functions in healthy elderly adults [20]. Studies suggest a positive effect of physical activity and structured exercise on cognitive functions, with the most consistent findings being reported for executive functions [20]. Video games that require the patient to perform physical movements while conducting cognitive exercises showed great potential for cognitive rehabilitation [15, 20]. An example of this approach is “X-Torp” (on Microsoft R Kinect), an action serious game played with the Kinect in which the player controls a submarine in real-time with his/her stationary movements involving mainly the lower limbs. This game aims to destroy other ships in the sea and to accomplish short missions following a story plot. In a clinical study, “X-Torp” showed to enhance cognitive functions and physical activity [15]. Despite their potential effectiveness for neurological rehabilitation, the use of games with violent content is questionable and should be addressed carefully. However, a recent study showed that violent video games reduce child-to-parent violence rates [52] and longitudinal studies do not support a substantive long-term links between aggressive game content and youth aggression [53].

Moreover, combining motor and cognitive training could be useful due to the transfer effect between motor and cognitive skills [14]. In recent years, virtual reality-based DTx have been increasingly applied to the rehabilitation of patients with Parkinson's disease. The efficacy of virtual reality-based training was explored in a systematic review and meta-analysis of over a thousand participants [14]. The authors revealed that virtual reality training improves clinical outcomes in patients with Parkinson's disease, such as cognitive function, motor function, balance, coordination, and quality of life [14]. Exergames require the user to perform physical movements while conducting cognitive exercises and, therefore, have shown great potential for Parkinson's disease rehabilitation. Numerous randomized controlled trials were performed in the last 10 years, and most of them focused on using the “Microsoft Kinect” and the “Wii Balanced Board” for Parkinson's disease rehabilitation [14]. Further, Garcia-Agundez et al. [14] have revealed that in patients with Parkinson's disease exergame-based rehabilitation is feasible, effective, and safe.

The mechanisms to explain the effects of physical exercise on cognition are far to be completely known; however, it is supposed that motor exercise performance involves executive control of attention, largely controlled by the pre-frontal cortex. Therefore, it has been hypothesized that physical training may improve the efficiency of neural processes also involved in cognitive functions [20].

## Other neurological symptoms

Beyond sensorimotor and cognitive impairment, DTx could address other neurological symptoms (visual dysfunction, speech impairment, dysphagia, fatigue, depression, and pain) occurring in neurological disorders [54–67].

### Visual field impairment

A few studies have explored the feasibility and effectiveness of DTx for the treatment of visual impairment after stroke and the developed DTx mainly addresses visual field defects. Traditional rehabilitation therapy for visual field defects is based on a different approach: compensation therapy that focuses on intact residual abilities and restitution therapy which aims to regenerate the plasticity of neural tissue presenting repetitive light stimuli in the border zone between the blind field and the spared field [8]. “NeuroEyeCoach” and “VISIOcoach” are an example of digital compensation therapy and have shown to be effective computer-based compensatory therapies in patients with visual field defects [54, 55]. Visual perceptual learning has been introduced recently as restoration therapy. Huxling K et al. showed that visual discrimination training improves performance in visual field tests (Humprhey perimetry) in trained chronic stroke patients with cortical blindness compared with untrained patients [56].

### Dysarthria and dysphagia

Digital solutions to manage dysarthria and swallowing problems are lacking. Previous research has shown the beneficial effects of swallowing exercises combined with myofunctional tongue-strengthening therapy [57]. A study explored the immediate and long-term maintenance effects of 8-week home-based tongue exercises delivered through mobile health technologies on 12 elderly adults who complained of swallowing difficulties (i.e, increased aspiration rate and foreign body sensation in the throat) [57]. The intervention aimed at improving the swallowing tongue pressure in elderly adults. The app included a swallowing monitoring and intervention protocol with 3 therapy maneuvers: effortful prolonged swallowing, effortful pitch glide, and effortful tongue rotation. The participants demonstrated a significant increase in swallowing tongue pressure. However, long-term maintenance effects at 12 weeks post-intervention were not observed [57]. Further, a limited number of studies with the “Lee Silverman Voice Treatment” showed positive effects on swallowing function, voice quality, speech intelligibility, and hypomimia [58]. A study on patients with Parkinson's disease compared, the differences in recorded speech variables between people treated with conventional ‘in person’ “Lee Silverman Voice Treatment” and those treated remotely via iPad-based “Facetime”. Study findings showed that iPad “Lee Silverman Voice Treatment” is non-inferior to the traditional in person “Lee Silverman Voice Treatment” [58].

### Fatigue

Following a user-centered design and evidence-based process, a mobile health solution called “More Stamina” was created to help patients with multiple sclerosis to manage their fatigue. An ongoing trial is exploring the feasibility, acceptability, and usability of “More Stamina” [59]. It is a gamified task organization tool aiming to help people with multiple sclerosis managing their energy, minimizing the impact of fatigue on their daily life. The tool acts as a to-do list where users can input the task they want to accomplish that day [59].

### Depression

Another common symptom is depression and only an adequate treatment would improve the quality of life of patients.

An example of DTx to manage depression is “Deprexis” [60]. It is a Web-based self-help program that combines cognitive-behavioral therapy with a mobile platform and dialog technology. In a 9-week randomized controlled trial, its efficacy was explored in multiple sclerosis patients with depression. Subjects were randomized into the intervention group or into the control group (remaining on a waiting list). Study findings revealed an improvement in Beck Depression Inventory scores for the intervention group and a worsening in the control one, highlighting the efficacy of Web-based intervention programs for depression management in patients with multiple sclerosis [60].

In people with dementia reminiscence therapy has been used in many long-term care facilities [61]. Reminiscence therapy involves recalling positive events, people, and places from their past lives [61]. A software called “ReminX” to mitigate agitation and depression symptoms associated with Alzheimer's disease was developed [62]. “ReminX” allows uploading pictures and narration to create slideshow stories that depict essential moments in the patient’s life. A clinical proof-of-concept study was performed on fourteen patients. Results indicated that the software led to an immediate and significant decrease in anxiety, depression, and overall emotional distress after having viewed their story [62].

### Pain

Virtual reality has also been shown to be effective in reducing pain intensity and discomfort in patients with different types of chronic pain; it provides an intervention based on a distraction technique because it draws attention away from the patients' mental processing, thereby decreasing the amount of pain consciously experienced [63].

A recent study explored the effectiveness of a 3D head-mounted virtual reality tool to reduce neuropathic pain in people with spinal cord injury compared to using a 2D screen device. Sixteen men with established spinal cord injury and chronic neuropathic pain participated in a single-session randomized cross-over trial. Participants reported significantly lower pain intensity after the virtual reality intervention compared to 2D screen application suggesting that that immersive virtual reality could be a helpful adjunct to current pharmacotherapy [63].

A further virtual reality-based approach, based on the "referred sensation" phenomenon, was explored for patients who suffer from phantom limb pain [64]. People suffering from phantom limb pain can experience tactile stimuli applied to the cheek on their affected side as if it were coming from their phantom limb [64]. In a clinical study exploring this approach, nine participants with phantom upper limb pain performed virtual reality neurorehabilitation exercises in which they repeatedly touched a target object with a virtual representation of their affected limb. A tactile feedback to their cheek was applied when their virtual affected limb touched the target object. Two control conditions were included: tactile feedback was either applied to their intact hand or not applied at all. Study results showed that the analgesic effect of neuro-rehabilitative visual feedback during phantom limb movement was improved by applying somatosensory feedback to the cheek on the affected side [64].

A few studies have evaluated the efficacy of virtual reality-based proprioception rehabilitation for patients with pain. Reazei et al. [65] investigated a new virtual reality-based gaming tool called “CERVIGAME”, designed to reduce pain and improve proprioceptive function in patients with neck pain. Forty-four participants with nonspecific chronic neck pain were randomly assigned to virtual reality training or conventional proprioceptive training. Both groups completed 8 training sessions over 4 weeks. There were significant improvements in all variables in both groups immediately after and 5 weeks after the intervention. The results indicated that virtual reality training was more effective in reducing pain and disability compared to conventional proprioceptive training. [65].

## Therapy adherence and self-care management

DTx interventions could be beneficial in increasing medical adherence, maintaining healthy lifestyles, facilitating access to care, improving the effectiveness of care, and lowering costs of care [67–77].

Adherence to medical therapy in stroke patients remains an unmet need and continues to burden the healthcare system [66–68]. “FARMALARM” is a smartphone application available in the Spanish language used in secondary stroke prevention. It allows the patient to monitor his physical activity, record and share vital parameters and capillary blood sugar levels with the physician, monitor therapy adherence, and receive advice about a healthy lifestyle. Through visual and audible alerts, the app showed a valuable tool in secondary stroke prevention, improving medication adherence, and maintaining healthy behaviors [68]. Study findings disclosed a higher rate of total control of critical vascular risk factors in the group that followed the “FARMALARM” program compared to patients who did not [68]. In a pilot study, [67] a digital self-management program, using a personal coach and digital platform, was developed to improve the monitoring of vascular risk factors after stroke. The patients could record data regarding cardiovascular risk profile, through the platform. If the values added by the patients exceed the defined threshold, the platform sent an alert to the stroke coach. The vascular risk, assessed with the “Systematic COronary Risk Evaluation: High and Low cardiovascular Risk Charts”, was significantly reduced at 6 months in the intervention group (as compared to baseline scores) [67].

For patients with multiple sclerosis achieving adherence to long-term treatment with injectable disease-modifying drugs is challenging and mainly related to patient factors and satisfaction with both medication and application systems [69]. Since higher satisfaction with a device may positively impact adherence, a platform-based approach for a complex treatment regimen, allowing for monitoring of the disease and treatment course, as well as the interaction between the patient and the neurologist, may offer an opportunity for both patients and physicians/nurses to improve their understanding of the patients’ condition and ultimately to foster adherence [69]. To date, numerous smartphone applications are available for self-managing of medications and injections in multiple sclerosis. The most frequent characteristics observed in these subcategories of apps include: entering medication names, recording missed medication dosages, managing injections, tracking injection sites, reporting and sharing data, notifying the end of medication stock, and accessing pharmaceutical information [69].

“MS DIALOG” is a web and mobile-based software application that captures data on self-administration of subcutaneous interferon β-1a, clinical outcomes, and patient-reported outcomes in patients with multiple sclerosis outside the clinic [70]. In a study exploring its usability among patients and clinicians, “MS dialog” was considered easy to use and superior to patients’ previous methods for improving self-management of their condition [70]. A more structured digital tool to manage therapy in multiple sclerosis is “BETACONNECT”. The “BETACONNECT” system is a platform-based approach to monitor disease-modifying therapy [71]. It combines auto-injector technology with digital tools to support patient self-management and facilitate communication between patients and healthcare providers. The system encompasses an injector and an app for the patients and a platform for the nurse and physician. The app facilitates individual management of IFN-β-1b therapy. It features injection planning and recording as well as an injection site rotation scheme. Patients may choose to be reminded by email or push message when the next injection is due. To enhance communication, patients can choose to share their injection data with their multiple sclerosis nurse or physician. In this context, the “BETACONNECT” offers a unique benefit since it automatically records every injection, thus permitting a largely unbiased evaluation of adherence [72]. On the contrary, a study exploring the usefulness and validity of a smartphone-based e-diary to promote the adherence to therapy in MS patients concluded that smartphone reminders did not significantly improve the medication possession rate of disease-modifying therapies [72].

Optimal medical compliance and satisfactory seizure control are still unmet needs in people with epilepsy [73]. One of the first tools for delivering self-management content is the “WebEase” platform. It encompasses three modules (medication adherence, sleep, and stress) that patients with epilepsy could complete online. In a clinical trial, the platform showed to improve adherence, self-management and to reduce the stress level in the intervention group compared to the control group [73]. Consistently, a recent clinical trial on 327 adult epilepsy patients explored whether an intervention based upon a smartphone app would improve self-management and seizure control. Participants were stimulated to improve their therapeutic compliance and lifestyle to reach seizure control and optimal quality of life. The app provided patients a multi-faced digital assistance encompassing medication calendar, online educational forums and blogs, a facility for prompt online reporting of seizures and consultations, and online surveys. The study provided evidence for the benefits of epilepsy-specific apps in improving patient self-management and in reducing seizure frequency [74].

Further non-pharmacological interventions, such as cognitive therapy, psychosocial and educational interventions, also showed to reduce epileptic seizures. “Epicadance” is a developing mobile medical app for patients with epilepsy that integrates into mobile software all these therapeutic modalities combining epilepsy self-care, behavioral interventions, medication reminders, and anti-seizure music, such as Mozart’s sonata K.448 [75].

A further digital solution to improve treatment adherence and self-care was explored in the recent trial [76]. In this study, patients in the intervention group downloaded the “Parkinson Tracker App” and the control group underwent conventional treatment. Through the app, data on the following self-monitoring measures were collected: sleep, exercise, balanced diet, mood, energy, medication, and movement. Compared to conventional treatment, “Parkinson Tracker App” significantly improved the short-term self-reported medication adherence and patients’ perception of the quality of clinical consultation [76].

Finally, some wearable sensors such as accelerometers or electromyographic recordings have been developed to detect motor seizures in people with epilepsy [77]. An example is “Epi-Care free”, a wireless wrist accelerometer that records generalized tonic–clonic seizures with high sensitivity (90%) and a low rate of false alarms (0.1/day) similar to what has been determined in epilepsy monitoring units [77].

Self-care and disease management in the home environment also includes the potential use of on-skin wearable technologies (temporary or permanent tattoos) that worked as a touchpad or even stored and transmitted health information [78]. The tools-based devices, which are very flexible and non-permanent, may overcome limitations related to the monitoring of EEG in a lab or clinic, allowing the long-term non-invasive recording of brain signals while people are out of the lab and moving about [79]. Furthermore, real-time monitoring of respiration through transthoracic impedance measurements has been also investigated using unperceivable temporary tattoo electrodes [80]. This technology has been shown to be suitable for real-time monitoring of respiration but also for real-time monitoring of different bio-electric signals, such as EMG and ECG signals.

Self-management of migraine in adolescents is complex and has important implications for health outcomes. “MIGRAINE MANAGER'S” is a digital therapeutic self-management tool for adolescents with migraine [81]. A single-arm open-label trial evaluated the efficacy of “MIGRAINE MANAGER'S”. A significant decrease in headache days from 17 at baseline to 8 at 2 months/8 weeks was experienced by patients. Moreover, patients reported an improvement in the patient physical functioning quality of life [81].

The randomized controlled trials and randomized cross-over studies on DTx in Neurology, discussed in this review, are summarized in Table 1.

**Table 1:** Summary of randomized controlled trials and randomized cross-over studies on DTx in Neurology

Authors (Reference number)	Title	Intervention area	Tool	Percentage of patients that completed the study	Primary outcome reached	Strengths	Limitations
Subramanian et al. 2013 [9]	Arm Motor Recovery Using a Virtual Reality Intervention in Chronic Stroke: Randomized Control Trial	Stroke	Virtual reality platform	100% of patients completed the study in both groups	Yes	Randomized controlled trial design Type of intervention	Small sample size
FernándeznoGonzalez et al. 2019 [10]	Leap motion-controlled video game based therapy for upper limb rehabilitation in patients with Parkinson’s disease: a feasibility study	Parkinson disease	Leap motion controlled system	23 out 26 patients initially selected (88%) were enrolled. 100% completed the study	Yes	Randomized controlled trial design Type of intervention	Selection bias Sample size
Kalron et al. 2016 [13]	The effect of balance training on postural control in people with multiple sclerosis using the CAREN virtual reality system: a pilot randomized controlled trial	Multiple sclerosis	Virtual reality platform	93% of patients completed the study in both groups	Yes	Randomized controlled trial design	No follow up Small sample size
Cannell et al. 2018 [16]	The efficacy of interactive, motion capture no based rehabilitation on functional outcomes in an inpatient stroke population: a randomized controlled trial	Stroke	Motivating virtual reality platform	87,5% and 95% completed the study, respectively in the intervention group and control group	Yes	Randomized controlled trial design	Small sample size Short follow up
Yazgan et al. 2019 [17]	Comparison of the effects of two different exergaming systems on balance, functionality, fatigue, and quality of life in people with multiple sclerosis: a randomized controlled trial	Multiple sclerosis	Exergaming system	93% in the Nintendo Wii Fit group, 75% in the balance trainer group and 100% in the control group completed the study	Yes	Randomized controlled trial design	Possible selection bias No comparison with conventional rehabilitation method Short follow up
Liao et al. 2014 [18]	Virtual reality-based training to improve obstacle crossing performance and dynamic balance in patients with Parkinson’s Disease	Parkinson’s disease	Virtual reality platform	100% in the Virtual reality group, 100% in the active control group and 91% in the passive control group completed the study	Yes	Randomized controlled study	Small sample size Short Follownoup
Ribas et al. 2017 [19]	Effectiveness of exergaming in improving functional balance, fatigue and quality of life in Parkinson's disease: a pilot randomized controlled trial	Parkinson’s disease	Exergame	100% completed the study in both group	No	Randomized controlled study	Small sample size
Ozdogar et al. 2020 [21]	Effect of video-based exergaming on arm and cognitive function in persons with multiple sclerosis: A randomized controlled trial	Multiple Sclerosis	Videonobased exergaming	95% in the exergaming group, 89% in the conventional rehab group and 100% in the control group (no intervention) completed the study	Yes	Randomized controlled trial design with active and passive control group Type of intervention	Short follownoup Only clinical outcome measures
McEwen et al. 2014 [22]	Virtual reality exercise improves mobility after stroke: an inpatient randomized controlled trial	Stroke	Virtual reality training	74 out 91 patients initially selected (88%) were enrolled. 84% and 58% completed the study, respectively in the intervention group and in the control group	Yes	Randomized controlled trial design Type of intervention	Follow up length
Frevel et al. 2015 [25]	Internet-based home training is capable to improve balance in multiple sclerosis: a randomized controlled trial	Multiple sclerosis	Internet-based home training	88% of patients completed the study in both groups	Yes	Type of intervention Randomized controlled trial design	Small sample size Absence of control group Different duration of intervention in the 2 groups
Palmer et al. 2012 [32]	Computer therapy compared with usual care for people with longnostanding aphasia poststroke a pilot randomized controlled trial	Aphasia	Computer therapy	76% and 64% completed the study, respectively in the intervention group and control group	Yes	Randomized design Type of intervention	Single blinded study Randomization method Tool not validated
Marshall et al. 2018 [38]	Technology-enhanced writing therapy for people with aphasia: results of a quasi-randomized wait list controlled study	Aphasia	Dragon and WriteOnline no computer software for writing therapy	73% and 100% completed the study, respectively in the intervention group and control group (delayed intervention)	Yes	Quasinorandomized design Type of intervention	Short follow up Type of comparison Small sample size Selection bias (patients younger than most stroke survivors)
Pedullà et al. 2016 [40]	Adaptive vs. non-adaptive cognitive training by means of a personalized App: a randomized trial in people with multiple sclerosis	Multiple sclerosis	Mobile application	100% of patients completed the study in both groups	Yes	Randomized trial nature Type of intervention	Partecipants differences at baseline Limited test used at follow up Not excluded the dropnoout at follow up
Bove et al. 2020 [41]	A novel in home digital treatment to improve processing speed in people with multiple sclerosis: a pilot study	Multiple sclerosis	Home digital treatment	86% and 95% completed the study, respectively in the intervention group and control group	Yes	Double blind randomized control trial Type of intervention Sample representative of MS population Low cost and low risk	Heterogeneity on MRI parameters
Robert et al. 2020 [47]	Efficacy of a Web App for Cognitive Training (MeMo) Regarding Cognitive and Behavioral Performance in People With Neurocognitive Disorders: Randomized Controlled Trial	Neurocognitive disorders	Web application	88% and 91% completed the study, respectively in the intervention group and in the control group	No	Randomized controlled trial design Type of intervention	Small sample size Possible selection bias
Meyer et al. 2009 [60]	Effectiveness of a Novel Integrative Online Treatment for depression (Deprexis): Randomized Controlled Trial	Depression	Web-based program	49% and 75% completed the study, respectively in the intervention group and control group (delayed intervention)	Yes	Randomized controlled trial Type of intervention	High attrition rate Heterogenous sample of users Possible selection bias (only people comfortable with computer technology) Lack of multimedia components
Austin et al. 2020 [63]	The short-term effects of head-mounted virtual-reality on neuropathic pain intensity in people with spinal cord injury pain: a randomised crossover pilot study	Pain	Virtual reality platform	100% of patients completed the study in both groups	Yes	Randomized crossnoover design Type of intervention	Small sample size Heterogenous sample according to prescribed drugs Focused on short term outcomes
Rezaei et al. 2019 [65]	A Novel Virtual Reality Technique (Cervigame®) Compared to Conventional Proprioceptive Training to Treat Neck Pain: A Randomized Controlled Trial	Pain	Virtual Reality Technique	95% of patients completed the study in both groups	Yes	Randomized controlled trial design Type of intervention	Only clinical outcome Short follow up
DiIorio et al. 2011 [73]	Results of a research study evaluating WebEase, an online epilepsy selfnomanagement program	Epilepsy	Online self-management program	72% and 81% completed the study, respectively in the intervention group and control group	Yes	Randomized design	Possibile selection bias Social desirability biases
Si et al. 2019 [74]	Optimising epilepsy management with a smartphone application: a randomised controlled trial	Epilepsy	Mobile Application	92% and 82% completed the study, respectively in the intervention group and in the control group	Yes	Randomized controlled trial design Population size	Selection bias Information bias Follownoup length Focus on medication and not on psychological features
Lakshminarayana et al. 2017 [76]	Using a smartphone-based self-management platform to support medication adherence and clinical consultation in Parkinson’s disease	Parkinson’s disease	Mobile application	64% and 82,5% completed the study, respectively in the intervention and control group	Yes	Randomized controlled trial design Multicenter design	Short follow up

## Conclusions

The available data suggest that DTx can be applied to support traditional care of several neurological dysfunctions.

However, there are some open issues to be addressed: (1) the economic impact on the healthcare system; (2) the limited validation of the digital devices in non-English languages; (3) the lack of standardized protocols of intervention.

Moreover, in less developed areas, the lack of high-speed broadband access may account for the inability to reach underserved populations.

Remarkably, with only one exception (“FARMALARM®”, available in Spanish), all DTx devices identified in this article were available only in the English language.

Concerning virtual reality-based and video game-based tele-rehabilitation there is no consensus about the outcome measures, the training duration/ intensity, and the types of the exergame to be played to assess the clinical effectiveness of an exergaming intervention.

A huge threat to the implementation in clinical practice of DTx is the current lack of regulation and reimbursement guidelines. Understanding and overcoming barriers to effective regulation and reimbursement of DTx is a key element to promote the use of DTx in clinical practice.

Future studies are needed to identify the best intervention protocols, efficacy, safety, feasibility and benefit–cost ratio to promote the use of reimbursed DTx devices in clinical settings.

In this regard, the lesson from COVID-19 pandemic has been that telemedicine and digital devices not only enabled remote consultation, but also simultaneous image transmission and information communication, which greatly reduced the pressure on frontline medical staff [82]. More efforts should be prompted in the future for promoting DTx services not only in epidemic conditions but also in routine care.

## Data Availability

Not applicable.
